# High Performance Full-Inorganic Flexible Memristor with Combined Resistance-Switching

**DOI:** 10.1021/acsami.2c02264

**Published:** 2022-04-27

**Authors:** Yuan Zhu, Jia-sheng Liang, Vairavel Mathayan, Tomas Nyberg, Daniel Primetzhofer, Xun Shi, Zhen Zhang

**Affiliations:** † Division of Solid-State Electronics, Department of Electrical Engineering, Uppsala University, Uppsala 75121, Sweden; ‡ State Key Laboratory of High Performance Ceramics and Superfine Microstructure, Shanghai Institute of Ceramics, Chinese Academy of Sciences, Shanghai 200050, China; § Department of Physics and Astronomy, Uppsala University, Uppsala 75121, Sweden

**Keywords:** flexible memristors, Ag_2_S films, resistance switching, Schottky barrier modification, silver filament

## Abstract

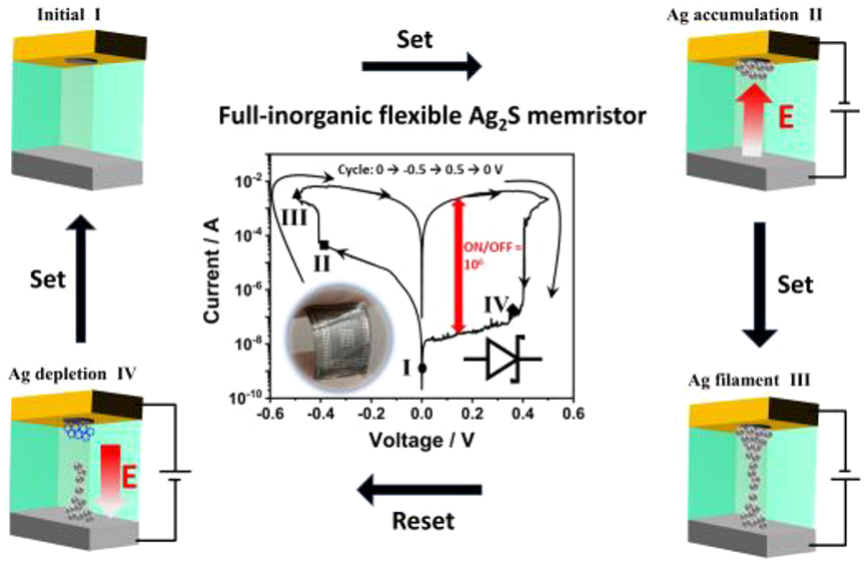

Flexible memristors hold great promise for flexible electronics applications but are still lacking of good electrical performance together with mechanical flexibility. Herein, we demonstrate a full-inorganic nanoscale flexible memristor by using free-standing ductile α-Ag_2_S films as both a flexible substrate and a functional electrolyte. The device accesses dense multiple-level nonvolatile states with a record high 10^6^ ON/OFF ratio. This exceptional memristor performance is induced by sequential processes of Schottky barrier modification at the contact interface and filament formation inside the electrolyte. In addition, it is crucial to ensure that the cathode junction, where Ag^+^ is reduced to Ag, dominates the total resistance and takes the most of setting bias before the filament formation. Our study provides a comprehensive insight into the resistance-switching mechanism in conductive-bridging memristors and offers a new strategy toward high performance flexible memristors.

## Introduction

Wearable electronics that work to read, store, and analyze biological information when interacted with human skin have attracted extensive research attention toward smart applications, such as electronic skin, artificial perception, health monitoring, etc.^1^ Considering that human skin has complex curved surface variations, especially under body movements, wearable electronics should accommodate the change of working environment by bending, to combine the data processing capability with device flexibility. As an important component in these electronics, flexible memristor (FM) is expected to store and process the information by switching between high and low resistance states on curved surfaces.^2−4^ In past decades, numerous efforts have been devoted to achieve the flexibility in memristors. Different dielectric and semiconductor thin films, in which the oxygen vacancy or metallic filaments can be formed to change the device resistance, were deposited on polymer substrates to fabricate the organic–inorganic hybrid flexible memristors, including the resistive random-access memory (RRAM),^5−7^ conductive-bridging memristor (CBM),^8^ etc. Full-organic FMs are also developed based on the intrinsically flexible organic resistance switching materials.^2,3^ However, the currently available full-organic FMs normally require high threshold voltage to switch resistance due to the slow mass transportation kinetics in organic electrolyte,^9,10^ while hybrid FMs suffer from short lifespan due to the inherent incompatibility between brittle inorganic electrolyte and flexible polymer substrate.^11−13^

Recently, our discovery of intrinsically flexible inorganic materials suggests a potential solution to the aforementioned problem. α-Ag_2_S is an intrinsically ductile semiconductor at room temperature with a carrier mobility of about 100 cm^2^V^–1^s^–1^ and a band gap of about 0.9 eV.^14,15^ It can simultaneously conduct both Ag^+^ ions and electrons under electric field^16^ and is a promising candidate as the functional electrolyte for FMs. The subsequent electrical bias-induced formation and destruction of Ag filaments within this material, has been utilized to build up rigid conductive-bridge memristors (CBMs) with conventional symmetric nanoscale cross-bar device structure.^17^ The reported ON/OFF ratio for these nano CBMs^18,19^ was limited to around 100, although higher switching ratio was demonstrated in a large millimeter-scale Ag_2_S memristor.^20^ Besides, the reported resistance-switching mechanism for Ag_2_S memristors only involves simplified filament formation/ablation processes, which could not fully account for the real memristor characteristics. High performance nanoscale Ag_2_S-based memristor devices, especially on flexible form, remain to be demonstrated. In this work, we fabricate full-inorganic nanoscale FMs, by using free-standing ductile α-Ag_2_S films as both flexible substrate and functional electrolyte. This nanoscale FM achieves ultrahigh performance together with room-temperature flexibility. A combined resistance switching mechanism behind the exceptional memristor performance is discovered. Based on this understanding, a new strategy in device design to achieve high performance FMs is also proposed.

## Results and Discussion

### Asymmetric Device Structure and Exceptional Behavior

**Figure 1:**
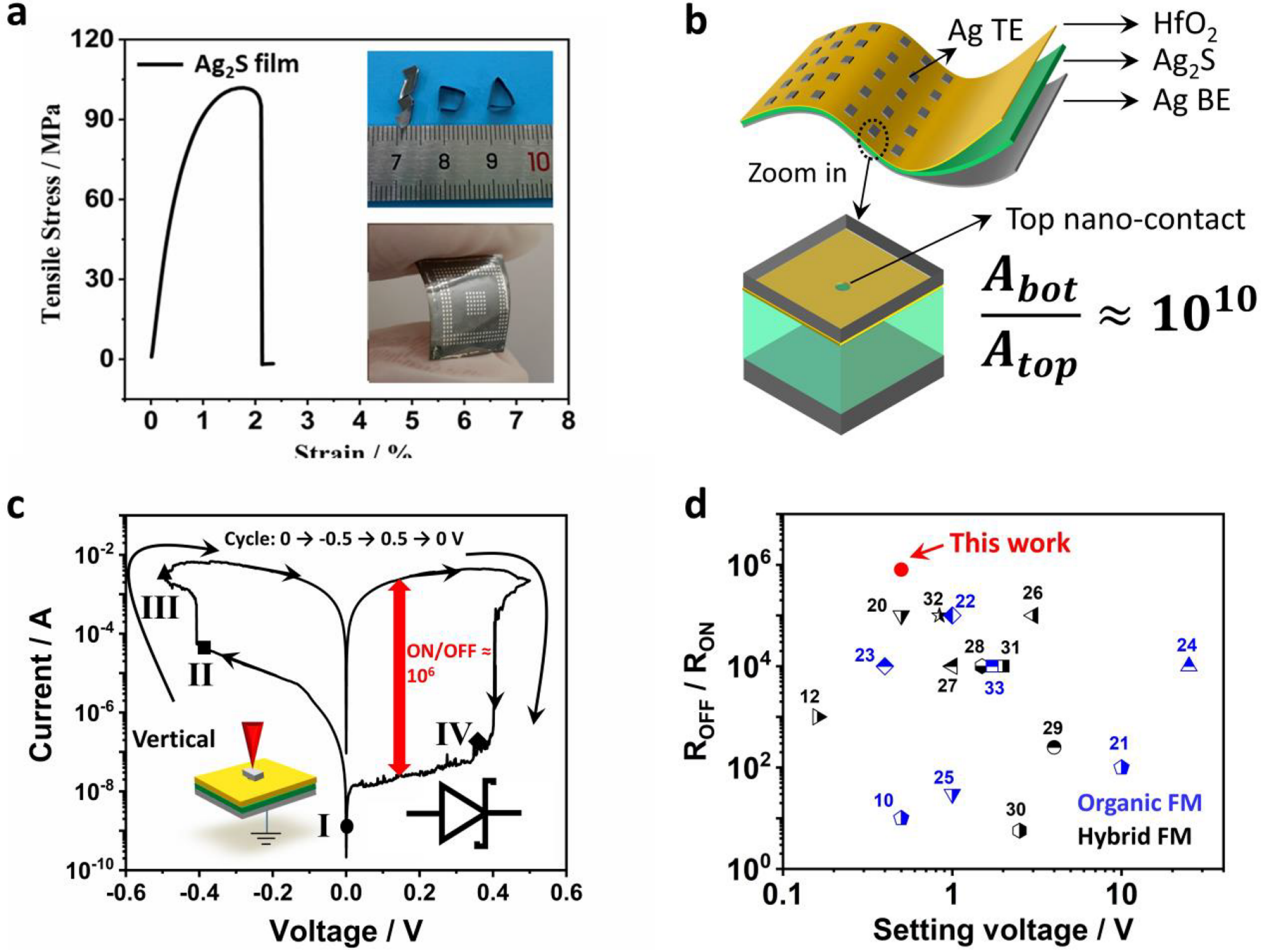
Demonstration of full-inorganic Ag_2_S flexible memristor. (a) Mechanical tension test of our Ag_2_S film at room temperature. The insets show bended flexible Ag_2_S film (upper) and Ag_2_S-based FMs (lower). (b) Schematic illustration of FM structure. The device consists of four layers (from top to bottom): silver top electrode (Ag TE)/HfO_2_ (dielectric layer for the nanoscale contact hole formation)/Ag_2_S electrolyte/silver bottom electrode (Ag BE). The size of the top contact via the nanohole (in HfO_2_ layer) is about 10^10^ times smaller than that of bottom contact (the dimensions shown are not scaled to the actual size). (c) Current (I)-voltage (V) characteristics of Ag_2_S-based memristor in vertical configuration. Different stages of the resistance switching processes are marked by I, II, III, IV in the curve. (d) Comparison of the ON/OFF ratio under different setting voltages between Ag_2_S-based memristor and the recently reported full-organic^10,17−25,33^ and hybrid^12,20,26−32^ flexible memristors.

The free-standing α-Ag_2_S films were roller-pressed from a bulk Ag_2_S ingot prepared by solid-state reactions (see Supporting Information (SI) Figure S1 for Ag_2_S film composition and phase analysis). The obtained 100 μm-thick Ag_2_S film endures an elongation of 2.1% under ∼100 MPa tensile stress (Figure 1a). The small tensile modulus value is comparable to some metallic materials and demonstrates its excellent mechanical strength and flexibility.^15^ Flexible memristors were fabricated directly on free-standing α-Ag_2_S films without any addition of polymer substrate in our device demonstration. Different from the conventional symmetric memristor device with cross-bar structure, here, an asymmetric device structure was used. The size of the nanoscale top contacts, formed in the 100 nm contact holes via the 5 nm-thick cap HfO_2_ layer (Figure 1b), is about 10 orders of magnitude smaller than the bottom contact (1 × 1 cm^2^ contact size) (see SI Figure S2 for device fabrication and a top-view scanning electron microscopy (SEM) image of the 100 nm contact hole array). This asymmetric contact structure ensures negligible contribution from the contact resistance of the bottom interface, if the device is measured in the vertical configuration between top and bottom contacts. The obtained device shows a typical bipolar memristor behavior (see Figure 1c) as demonstrated by the current (*I*)-voltage (*V*) characteristics recorded by scanning the voltage from 0 V→ −0.5 V → 0.5 V → 0 V on top electrode. The negative bias sets the memristor to a low-resistance state (about 10s of Ω at −0.5 V), which is well preserved until the device is reset back to a high resistance state (about 10 MΩ) with positive bias. The obtained ON/OFF ratio is about 10^6^, a record high number compared to the reported values of FMs (Figure 1d) under a low setting voltage.

### Reproducible and Stable Resistance Switching

**Figure 2:**
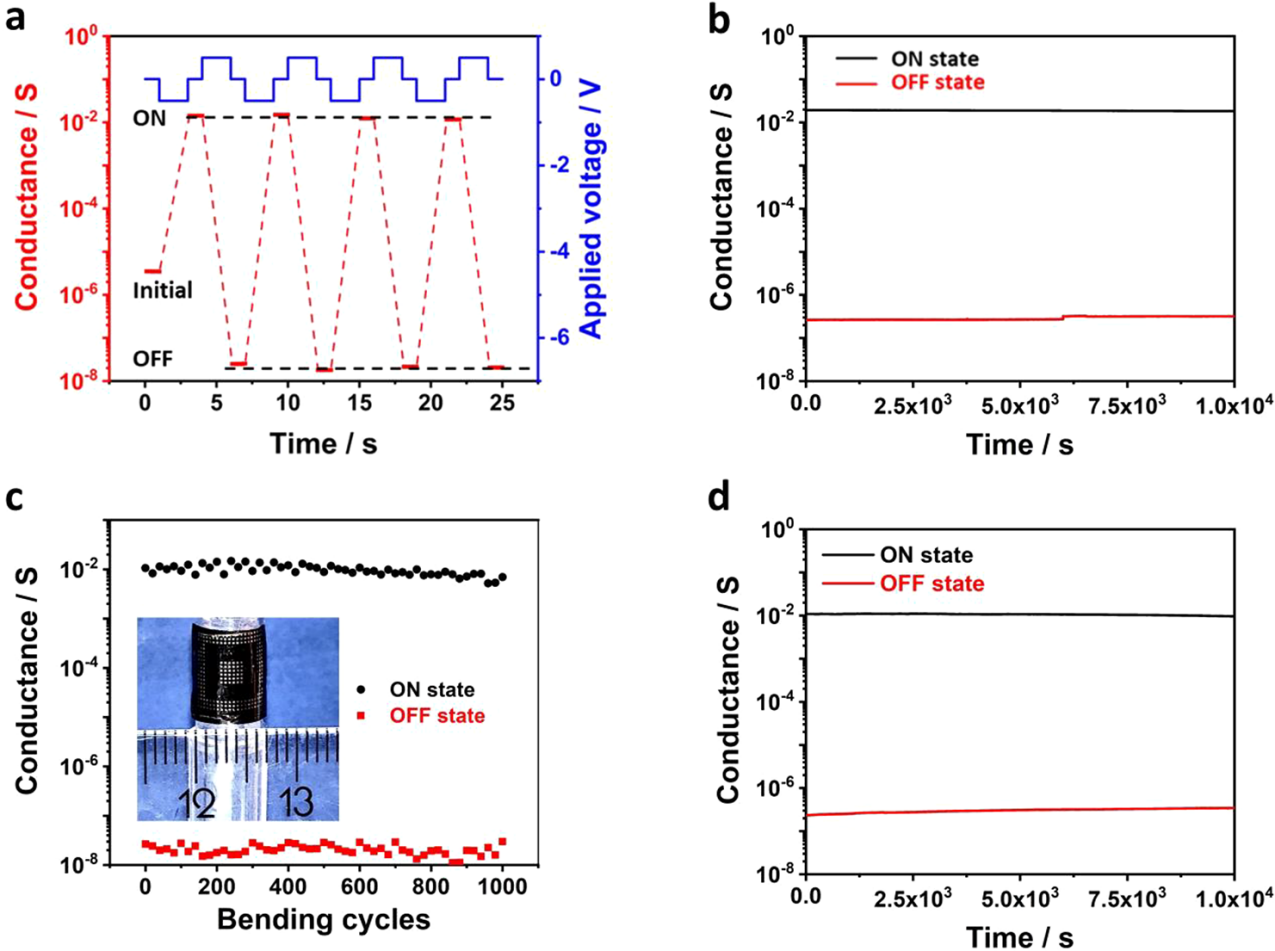
Data endurance and retention. (a) Conductance evolution under four cycles containing set (at −0.5 V), reset (at 0.5 V) and read (at 0.005 V) processes. (b) The data retention of ON and OFF states recorded in 10^4^ seconds. (c) Resistance variation before and after mechanical bending. After each bending (with 3 mm curvature radius as shown in the inset), the device was recovered to the flat state and its conductance versus bending cycles were recorded. (d) The in situ data retention recorded when the device was kept in bending state with 3 mm curvature radius.

To exam the switching stability, careful endurance tests were conducted. Under repetitive cycles of programmed set → read → reset → read pulse biases (Figure 2a), our FM shows reproducible switching with repeatable ON and OFF states. In further endurance test as shown in SI Figure S3, about 85% of 50 cycles result in the ON state conductance within (3 ± 1) × 10^–2^ S. We also characterized the device-to-device variation among 35 devices (see SI Figure S3d). The conductance of the ON and OFF states in all the devices falls in the narrow range of (1.5 ± 0.5) × 10^–2^ S and (1.5 ± 0.5) × 10^–8^ S, respectively, and the statistical distribution of ON/OFF ratio shows the most significant aggregation from 8 × 10^–5^ to 1 × 10^–6^. In addition, the long-term data storage is also demonstrated in Figure 2b, where the device conductance remains stable after 10^4^ s retention for the measured ON and OFF states, respectively. More importantly, these stable switching properties can be well maintained under deformation. We bent the device with the curvature radius of 3 mm, and recorded the conductance after recovering it to the flat state. As shown in Figure 2c, during the repeating 1000 bending tests, the conductance of the ON and OFF states was retained at their respective states level. Even if the device remained bent, it still maintained the long-term stability as indicated by the retention test shown in Figure 2d.

### Interface-Filament Combined Resistance Switching Mechanism

The exceptional ON/OFF ratio in our asymmetric device is obviously far beyond the conventional symmetric devices. In addition, the simplified filament formation/ablation processes proposed in the literature is not consistent with the experimental data in this study. Specifically, the exponential decrease of resistance before the sharp current jump at about −0.4 V (between stage I and II in Figure 1c) cannot be attributed to the filament formation. The filament ablation cannot explain the resistance increase when a positive bias is applied to an initial memristor device (without any filament) (SI Figure S4). In order to investigate the resistant-switching mechanism, we model the relevant resistances in our device. The Ag_2_S is a lightly doped n-type semiconductor and it forms Schottky contacts with both silver electrodes. At the initial stage of the vertical configuration measurement, the total device resistance (*R*_tot_) consists of three parts: contact resistances at the top (*R*_*c*, top_) and bottom (*R*_*c*, bot_) Schottky contacts as well as bulk Ag_2_S resistance (*R*_bulk_).^34,35^
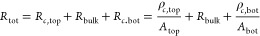
1

**Figure 3:**
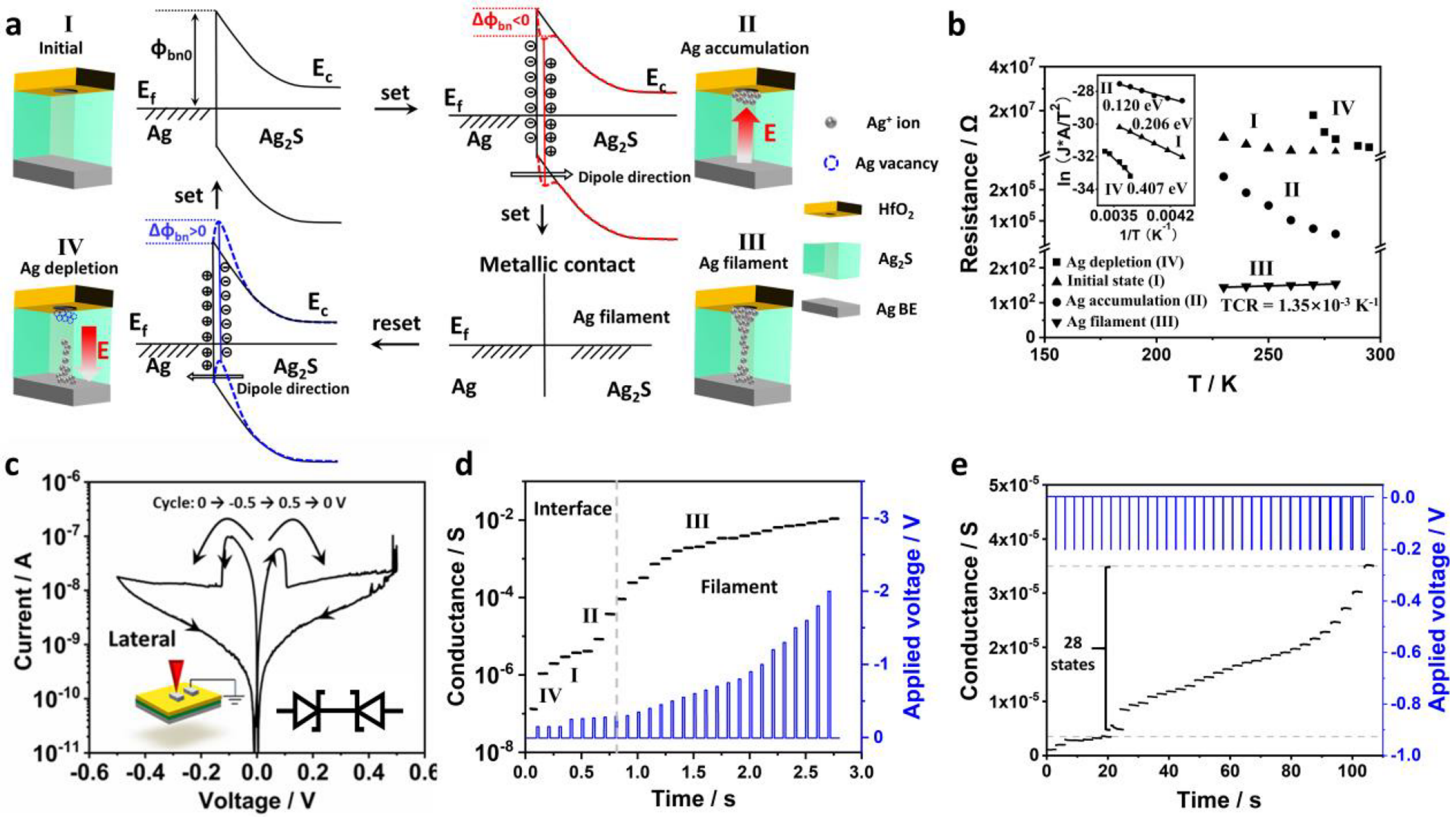
Interface-filament combined resistance switching mechanism. (a) Schematic illustration of resistance switching mechanism (The corresponding stages I, II, III, IV are also indicated in I–V curve in Figure 1c). The energy band at the interface of the top contact is modified by the accumulated positively charged interfacial Ag^+^ ions at stage II and negatively charged Ag vacancies at stage IV, resulting in SBH reduction and increase, respectively. The SBH reduction increases the conductance between stage I and II in Figure 1c. Subsequently, the formation of the Ag filament between the top and bottom electrodes with the increased set bias causes a sudden increase of the conductance between stage II and stage III. The ablation of the Ag filaments and SBH increase by reset bias decrease the conductance at stage IV. (b) Temperature dependence of the device resistance at initial (I), Ag^+^ accumulation (II), filament (III), and Ag^+^ depletion (IV) stages measured under low temperatures. The inset shows electron Schottky barrier height extraction for stages I, II, IV. (c) I–V curves recorded from a lateral device configuration between two neighboring identical 100 nm top contacts separated by a 200 μm distance. (d) Conductive states evolution (read at 5 mV) under pulse voltage stimulations (with 20 ms constant duration and variable amplitude from −0.15 V to −2.0 V). The obtained high density working states cross both interface and filament regions. (e) Conductive states evolution (read at 5 mV) under pulse voltage (with a fixed −0.2 V amplitude and variable width from 2 to 800 ms).

*R*_*c*, top_ and *R*_*c*, bot_ can be expressed by *ρ*_*c*_/*A*, where *ρ*_*c*_ is the contact resistivity and *A* is the contact area. *R*_*c*, bot_ is negligible due to its 10 orders of magnitude larger contact area compared to *R*_*c*, top_. Since the top Ag/Ag_2_S Schottky junction is reversely biased when applying the negative setting voltage, *R*_*c*, top_ overwhelms *R*_bulk_ in the setting process, as indicated by the drastic potential drop at top interface (see the simulated electric field distribution in SI Figure S5a). This is further experimentally confirmed by similar resistance switching behavior measured from Ag_2_S FMs with the same top/bottom Ag contacts but different Ag_2_S film thicknesses (SI Figure S6). Therefore, the measured resistance between stage I and II is dominated by the top Schottky contact, and its exponential decrease could be caused by the Schottky barrier modification. Indeed, the nanoscale top Schottky junction is reversely biased in the setting process and takes the most of the negative setting voltage. This results in an extremely strong electrical field of about 10^7^ V/m (SI Figure S5b) to accumulate Ag^+^ ions at top Ag/Ag_2_S interface, which leads to the formation of a strong interfacial dipole and reduces the electron Schottky barrier height (SBH), as schematically illustrated in Figure 3a (Ag accumulation - stage II). SBH modification based on space charge-induced interface dipole has been widely studied and used for contact optimization in silicon technology.^36,37^ Meanwhile, Ag^+^ ions are reduced to Ag atoms which grow toward the bottom electrode as filaments.^38^ The continuous filament will completely shunt both bulk Ag_2_S and contact resistances as it reaches the bottom electrode (see metallic contact stage III in Figure 3a). This is reflected by the sudden current jump at about −0.4 V, which is defined as the filament formation threshold voltage. The statistical distribution of threshold voltage in SI Figure S7b shows an aggregation below −0.2 V, owing to the residual filaments effect in the subsequent repeated scans.

Distinct temperature dependence of the resistance at stages I, II, and III strongly supports our hypothesis of SBH modification (Figure 3b). Stage I and II show a typical Schottky junction conduction behavior dominated by thermionic emission process (see the inset).^39^ The electron SBH is reduced from 0.206 to 0.120 eV from stage I to stage II. While stage III shows a typical linear temperature dependence of a metallic conductor (with a temperature coefficient of resistivity (TCR) of 0.00135 K^–1^), indicating continuous silver filaments formation. During the resetting process under positive bias, the formed Ag filaments are oxidized to Ag^+^ ions at the top electrode and get dissolved at about 0.4–0.5 V, resulting in a sudden drop of the current. Afterward, the strong local electrical field (SI Figure S5d) induced by the reformed top Schottky contact further drives away Ag^+^ ions and leaves negatively charged Ag vacancies at the top interface, which could bend the energy band upward and increase the barrier height, as illustrated in Figure 3a-IV. Low temperature measurement confirmed that the electron SBH was indeed increased to 0.407 eV after the reset process, which is responsible for the enlarged OFF state resistance beyond its initial value (shown in SI Figure S4). Increasing resetting voltage amplitude, even with small bias pulse, can increase OFF state resistance (see SI Figure S8) due to the further Ag^+^ depletion at the top interface. The low temperature characterization quantitatively confirmed the interface-filament combined resistance switching mechanism, where the sequential SBH modification and filament formation/ablation sum the resistance change to reach a large ON/OFF ratio.

To further verify this mechanism, our device was also measured under a lateral configuration between two identical 100 nm top contacts (see the inset in Figure 3c). This contact geometry is symmetric to emulate the conventional symmetric cross-bar structure. The obtained unipolar behavior shown in Figure 3c is the characteristic of the two identical back-to-back Schottky junctions. The initial resistance reduction of the reverse-biased cathode junction (induced by silver Ag^+^ accumulation) is overwhelmed by the drastically increased resistance of the anode junction (due to the Ag^+^ depletion) at about ±0.1 V bias. After that, the anode interface takes most of the voltage bias and thus limits the filament formation. The maximum ON/OFF ratio of the resulted butterfly shaped hysteresis loop is only ∼100, which is solely induced by interface resistance switching as also confirmed by the low temperature measurements (SI Figure S9). Our discovery also points out an important guideline to design high performance Ag_2_S memristors: The cathode junction, where the Ag^+^ is reduced to Ag, must dominates the total resistance and takes the most of the applied setting bias before the Ag filament formation. This strategy can be potentially realized in the conventional conductive-bridging structure by pairing the high resistance Schottky contact at the cathode interface with a low resistance ohmic contact at the counter anode interface, without using any large bottom electrodes.

The interface-filament combined mechanism also leads to densely populated conductive states in our memristor. As shown in Figure 3d, a series of writing pulses with fixed 20 ms constant duration but increasing amplitudes result in 10s of discrete nonvolatile states. This state evolution, containing silver depletion, accumulation, and filament formation, well reflects the electric bias-induced resistance switching processes at both interface and filament regions. Since the difference between the target and actual conductance could generate errors during memristor-based information processing, highly dense conductive states are greatly desired in memristors to ensure the accurate encoding from target conductance to conductive state.^40,41^ We show that in our flexible memristor, multiple-level conductive states are bias-dependent and further states evolution can be demonstrated by a more sophisticated pulse program. As shown in Figure 3e, small pulses (with a fixed −0.2 V amplitude but variable duration from 2 to 800 ms) further result in 28 states within 3.5 × 10^–6^ S to 3.5 × 10^–5^ S in interface resistance switching region, indicating the potential in continuous conductive states tuning. The retention test shown in SI Figure S10 demonstrates the nonvolatility of the 28 low conductance states in interface resistance switching region. Since their conductance is significantly lower than the filamentary conductive states, they hold the great potential for memristor array operations with much reduced power consumption. Furthermore, we show the synaptic plasticity containing the short-term potentiation (STP) and long-term potentiation (LTP), can be mimicked by controlling the applied pulse duration. As shown in SI Figure S11, nanosecond pulse induces typical STP behavior, while the nonvolatile conductive states excited by long pulses (above millisecond level) are LTPs. This indicates that our Ag_2_S memristors can be potentially utilized for neuromorphic computing in flexible artificial neuro network.

### Multiple-level Information Storage Demonstration

**Figure 4:**
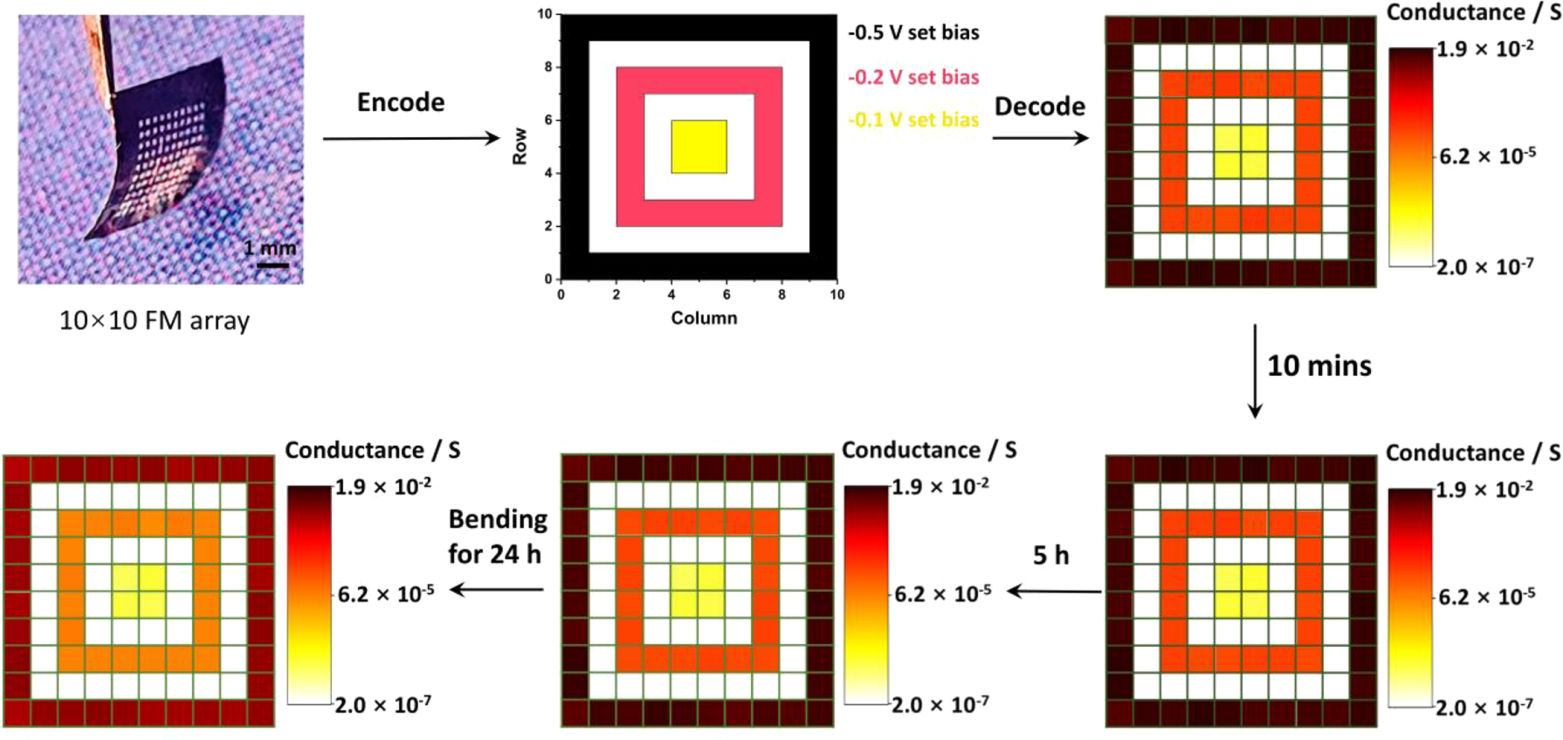
Information programming and retention demonstration for a 10 × 10 FM array. FMs in the array are reset to OFF state before three patterns are programmed into the array by setting the corresponding devices into three different conductance states. The conductance is read out and transformed to 256-level color-scale image to decode the stored pattern information. The information storage is further evaluated by recording the conductance evolution (read at 5 mV) after different silent treatments.

To demonstrate the ability of our device for basic information storage,^42^ we fabricated a 10 × 10 array of Ag_2_S based FMs. Before information compilation, these 100 FMs were all reset to OFF state as blank memory units. Three different patterns were programmed into memristors by respectively applying setting voltages with three different respective pulses (−0.1 V, −0.2 V, and −0.5 V amplitude with 1 s duration). The conductance of each FM in the array was read out and logarithmically mapped to a 256-level color-scale image between its maximum and minimum values. To test the information storage capability, the conductance evolution was monitored after different silent treatments (removing all the electric bias to the FMs for a certain period of time). As shown in Figure 4, the pattern information is well programmed and stored into three discrete operating states with conductance levels located at ∼10^–6^ S, ∼ 10^–4^ S, and ∼10^–2^ S level, respectively. After 5 h silent treatment, the device conductance only varied about 5% and the programed pattern was well preserved. To further demonstrate its capability as flexible memory cell, the array was bent for another 24 h with 3 mm curvature radius. Since the tiny silver filaments in memristor could spontaneously evolve to metallic nanospheres to minimize the interface energy between metallic filaments and active electrolytes, the conductance loss happens under the absence of external electric field.^43,44^ However, the programed pattern in our memristor array is still well preserved, despite some conductance decays over time. The demonstrated exceptional nonvolatility and promising fault-tolerance during data writing and reading of FM array are benefited from its large high ON/OFF ratio enabled by its device structure.

## Conclusion

We demonstrate free-standing full-inorganic Ag_2_S FMs with a record high 10^6^ ON/OFF ratio at small setting/resetting biases of ±0.5 V. It operates with high density nonvolatile states across 6 orders of magnitude in conductance. This extraordinary performance is attributed to the combined sequential resistance switching processes at the contact interface and filament regions, which can be realized either by an asymmetric contact geometry as demonstrated in this work, or by using a stable ohmic contact at the counter anode interface in a symmetric contact geometry such as conventional cross-bar structure. This study not only significantly advances the understanding of the resistance switching mechanism in conductive-bridging memristors, but also provides a promising strategy toward high performance FMs.

## Methods

### Ag_2_S Film Synthesis

Ag_2_S was prepared from high purity elemental Ag (99.999%, Alfa Aesar, shots), S (99.999%, Alfa Aesar, powders) with the stoichiometric ratio of 2:1. The stoichiometric admixture about 8 g was weighed out, sealed in an evacuated quartz tube, heated to 1000 °C, dwelt for 12 h before slowly furnace-cooled to 100 °C within 25 h. After that, the tube was annealed for 5 days at 450 °C to obtain the final product. The dense ingot was directly cut into pieces with the size of about 1 mm in thickness. After washed by acetone and ethanol, the as-prepared Ag_2_S pieces were directly roller-pressed into films with the different thickness. The feeding rate was ∼10 mm min^–1^, and the roller-pressing was consecutive until the film reached the target thickness (100 μm). The samples edge was trimmed during the rolling process. The obtained Ag_2_S is a lightly doped n-type semiconductor with resistivity about 40 Ω m and the effective doping concentration about 1.2 × 10^13^ cm^–3^.

### Ag_2_S-Based Memristor Fabrication

The Ag_2_S film was successively dipped in buffered Hydrofluoric acid (BHF), deionized water, acetone and isopropanol for surface cleaning. A 100 nm-thick blanket silver was then coated as bottom electrode layer by thermal-evaporation (with vacuum of 5 × 10^–6^ Torr and deposition rate of 0.1 nm s^–1^). On the top side, a 5 nm-thick HfO_2_ layer was first deposited at 170 °C by using atomic layer deposition (ALD). Nano contact holes were then patterned using e-beam lithography followed by reactive ion etching process (RIE) to etch through the HfO_2_ layer. Finally, 100 nm thick silver top electrodes were formed via the nano contact hole using a silver evaporation and lift-off process.

#### Characterization of Materials and Device

The Ag_2_S composition was analyzed by Rutherford backscattering spectrometry (RBS), where 2 MeV He^+^ ions were provided by a 15-SDH-2 5MV pelletron accelerator. X-ray diffraction (D8 ADVANCE instrument, Bruker) was used for phase characterization of polycrystalline Ag_2_S film. Tensile tests of Ag_2_S film were carried out in Dynamic and Fatigue Testing Systems (Z100, Zwick/Roell) with a loading rate of 1 mm min^–1^. Scanning electron microscope (SEM) images of nanohole array were collected from ASO2 Zeiss 1550 scanning electron microscope, where a small accelerating voltage of 1.0 kV was employed to avoid the degradation of Ag_2_S film under flood exposure of electron beam. Electrical characterization was conducted using Agilent B1500A Semiconductor Device Analyzer equipped with pulse/waveform generator. In bending tests, the initial conductance of both ON and OFF states is recorded when the device is flat. The device was then bended with 3 mm curvature radius and got recovered to flat state, followed with conductance reading (at 5 mV). This sequential process was conducted 1000 cycles and the conductance evolution versus the bending cycle was summarized in Figure 2c. Besides, the in situ device conductance measurement, where the device was kept bending (with the curvature radius of 3 mm) during the whole retention test, was also performed and the results were summarized in Figure 2d.

### Electric Potential and Electric Field Simulation

The electric field and potential distribution are simulated with standard Schottky junction model in COMSOL software. A sandwiched structure (100 nm top contact/100 μm-thick Ag_2_S electrolyte/1 cm^2^ bottom contact) is built according to the actual device dimension. The material properties were employed according to the database in COMSOL. The Poisson equation is solved by iteration with maximum iterations of 50 steps and error of 10^–7^. The electric field and potential distribution are calculated and replotted from the solution.

### Extraction of Electron Schottky Barrier Height

Low temperature measurement is conducted in cryogenic probe station equipped with liquid nitrogen supply system to extract the electron Schottky barrier height. The device was first cooled to liquid nitrogen temperature and then heated to room temperature. The resistance is read out by applying a 5 mV bias during heating process. The theoretical current density across an ideal Schottky junction follows eq 2:
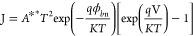
2

Here A** and *K* are the effective Richardson constant and Boltzmann constant, respectively.

eq 3 is obtained by taking the logarithm of eq 2:
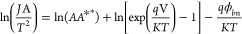
3

Where *A* is the contact area.

Since  passivates to the temperature variation at a 5 mV read voltage (the value only varies from −1.25 to −1.47 when temperature is increased from 230 to 280 K), eq 3 can be mathematically approximated to eq 4:
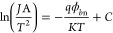
4

Where *C* equals to  and can be approximated to a constant.

Hence, by plotting  ∼ , one can extract electron Schottky barrier height from the slope of linear fitting (the slope equals to ).

## Supporting Information Available

Resistance switching process in lateral device configuration, X-ray diffraction pattern, and Rutherford backscattering spectrometry of Ag_2_S films, memristor device fabrication, and scanning electron microscope image of top nanocontact holes, device current under setting-resetting-reading process, the cycle-to-cycle and device-to-device variations of conductive states, resetting current trace of an initial memristor, simulated electric potential and electric field distribution of vertical configuration device using finite element analysis, resistance switching behavior of memristors fabricated on Ag_2_S films with different thickness, repetitive switching behavior of vertical and lateral structures, recorded device resistance at initial and OFF states after reset bias pulse, lateral device resistance (read at 5 mV) after setting at −0.1 V under low temperatures, data retention of conductive states in interface resistance switching region, and the post-synaptic current under 200 ns or 1 ms pulses (PDF)
