# Supplementary material for: High Performance Full-Inorganic Flexible Memristor with Combined Resistance-Switching
Source: ACS Appl Mater Interfaces. 2022 Apr 27;14(18):21173–80. doi: 10.1021/acsami.2c02264 (PMC9100493; doi:10.1021/acsami.2c02264)
Supplement: Supplementary file 1 — am2c02264_si_001.pdf [file am2c02264_si_001.pdf]

Supporting Information

High performance full-inorganic flexible memristor with combined resistance-switching

Yuan Zhu[1], Jia-sheng Liang[2], Vairavel Mathayan[3], Tomas Nyberg[1], Daniel Primetzhofer[3], Xun Shi[2] & Zhen Zhang[1]**

[1]Division of Solid-State Electronics, Department of Electrical Engineering, Uppsala University, Uppsala 75121, Sweden.

[2]State Key Laboratory of High Performance Ceramics and Superfine Microstructure, Shanghai Institute of Ceramics, Chinese Academy of Sciences, Shanghai 200050, China.

[3]Department of Physics and Astronomy, Uppsala University, Uppsala 75121, Sweden.

*Correspondence to: zhen.zhang@angstrom.uu.se (Z.Z.); xshi@mail.sic.ac.cn (X.S.)

Resistance switching process in lateral device configuration

The total resistance (R_{tot}) of lateral structure consists of three parts: bulk Ag_2S resistance (R_{bulk}), contact resistances of the left (R_{CL}) and right (R_{CR}) Schottky contacts. Since both contacts have an equal size, neither R_{CL} nor R_{CR} can be neglected. Our measurement starts with reversely biasing the left contact with negative bias, so R_{CL} initially dominates R_{tot} and takes most of the applied bias. The Ag^+ ions thus accumulate around the left contact and reduces R_{CL}. The measured current increases rapidly up to 10^{-7} A when the voltage bias is increased from 0 V to -0.1 V, which is very similar to the behavior of the vertical configuration within the same voltage range. Comparing to the top contact in vertical configuration, the effective bias applied on the left contact in lateral case is lower due to the potential drop on the nanoscale right contact. This leads to a lower current increasing speed of the later configuration. On the other hand, the right contact is forward biased and the electrical field around it will drive Ag^+ ions away. The resulted depletion of Ag^+ ions increases the Schottky barrier height of the right contact, which leads to the drastic increase of R_{CR}. Right contact resistance then dominates the total device resistance and drops the current at about -0.1 V. As a result, no filament is formed to bridge the two contacts in the whole 0 V - -0.5 V setting range. Indeed, no sharp current jump related to the filament formation is identified; and the maximum current level is about five orders of magnitude lower than the vertical configuration. The current transport process of lateral configuration device set at -0.1 V still shows thermionic emission characteristics (see in Figure S9). The same process happens when positive bias is applied, which leads to a symmetrical behavior compared with the negative bias branch. The

resulted butterfly-shaped hysteresis loop with the maximum ON/OFF ratio of ~100, is reproducible when the voltage scanning is repeated (Figure S7b). The result proves that the asymmetry in the contact sizes in the vertical configuration, which ensures nanoscale top junction dominates the total resistance and takes the majority of the setting bias, is crucial for the filament formation and the extra-high ON/OFF ratio. In contrary, the majority of the potential drop during the setting process with a symmetrical contact geometry (such as the conventional cross-bar structure) can shift from the cathode to the anode contact, which will in turn stop the Ag^+ ion accumulation and reduction at the cathode interface, thus preventing the filament formation.

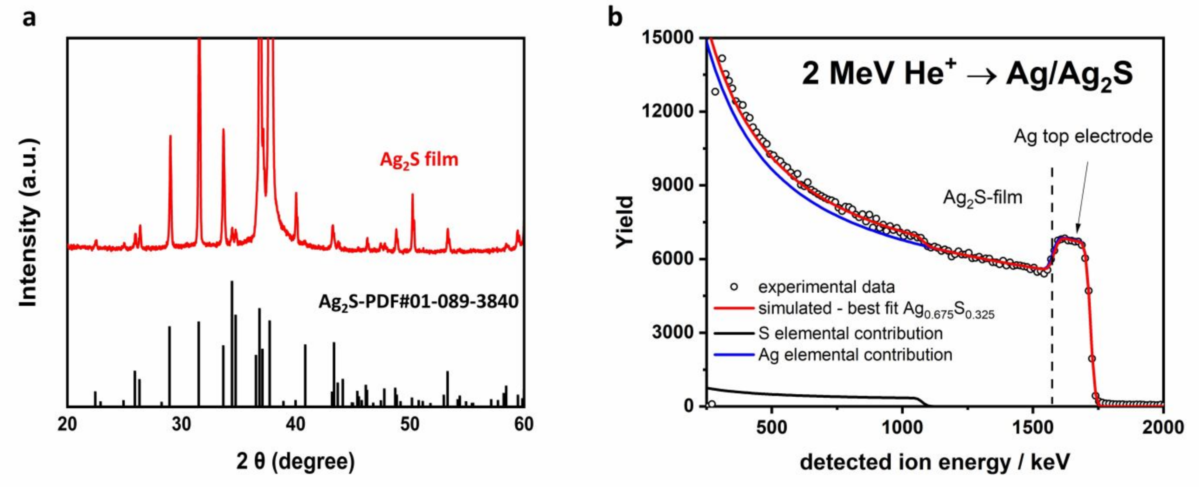

Figure S1. Ag_2S film characterization. (a) X-ray diffraction pattern of a 100 μm-thick Ag_2S film at room temperature, which is consistent with α-Ag_2S with Identification Card number of 01-089-3840. (b) Experimental RBS-spectrum recorded from an Ag_2S film (with an Ag top electrode as reference) using a microbeam of 2 MeV He^+ ions (open circles). The best fit to the experiment (red line) yields a composition of $Ag_{0.675}S_{0.325}$, which is close to the target stoichiometry of Ag_2S.

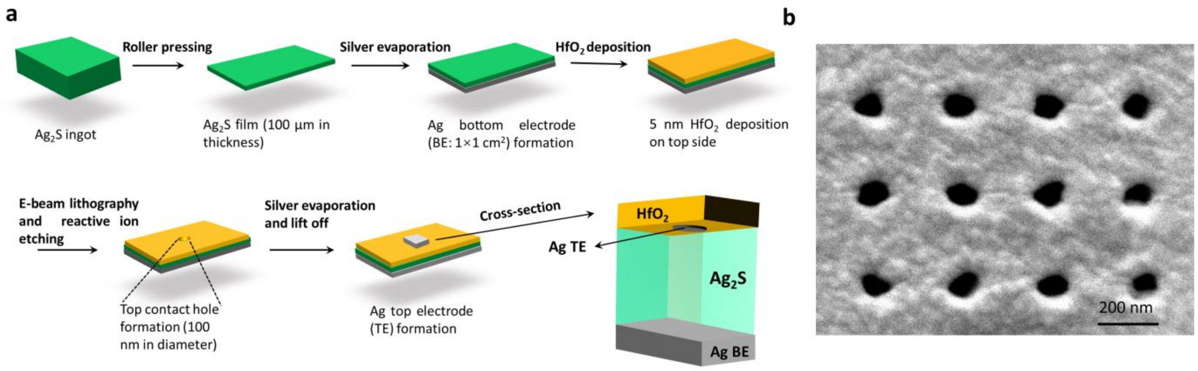

Figure S2. Device fabrication and characterization. (a) Process flow of Ag_2S-based memristor device fabrication. The dimensions shown are not scaled to the actual size. (b) Top-view scanning electron microscopy image for a 100 nm nano-hole array fabricated on top side of α-Ag_2S film. In each memristor device, only one contact hole is formed for top contact.

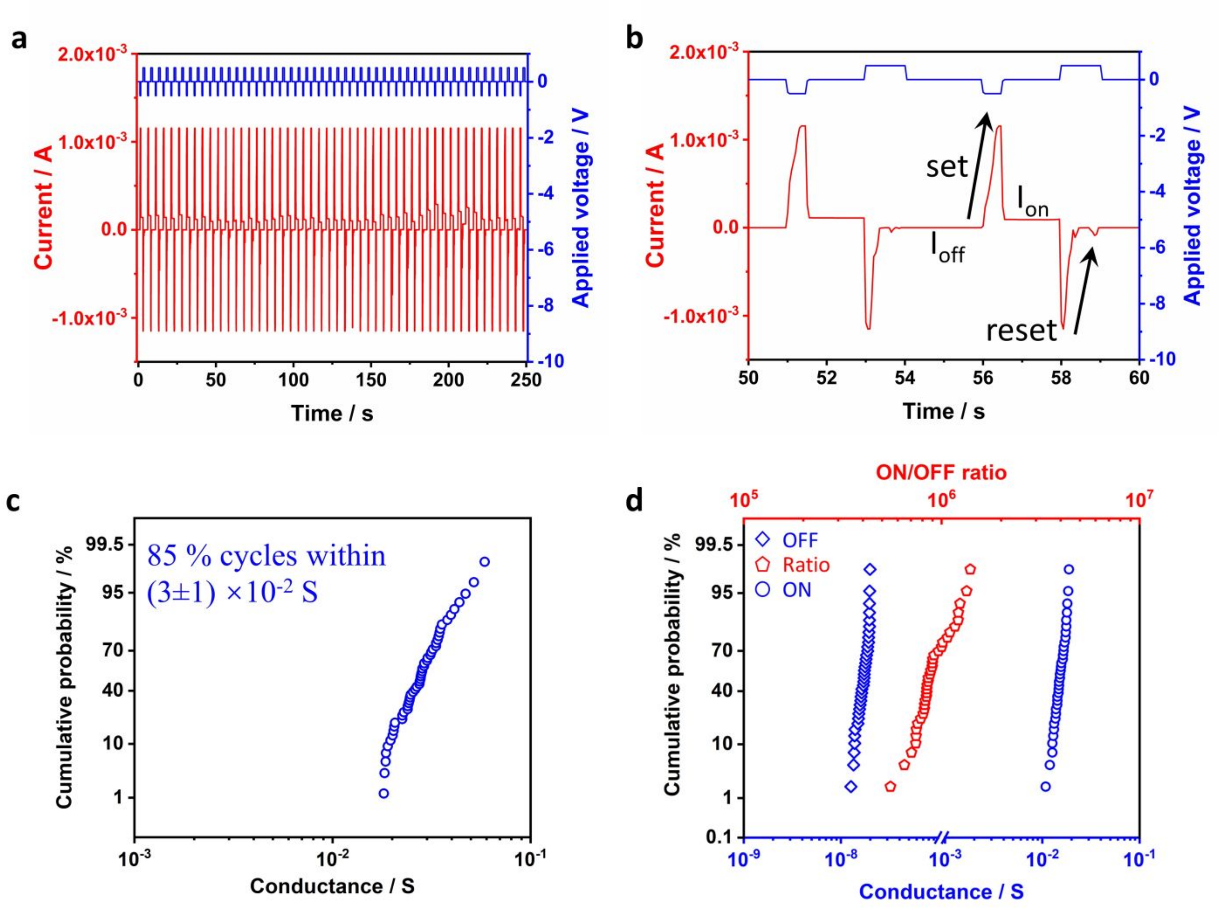

Figure S3. The current traces (with compliance at 1.15 mA) recorded with 50 repetitive voltage pulses. (a) 50 repetitive pulses, which contains setting (at -0.5 V), reading (at 5 mV) and resetting (at 0.5 V) were applied to the top electrode and the device current was recorded. (b) The detailed current response under setting and resetting bias demonstrates reproducible resistance switching behavior during the recorded cycles. (c) The cycle-to-cycle variations evaluated by analyzing the ON state current (read at 5 mV) after each setting process. (d) The device-to-device variations of conductive states and ON/OFF ratio based on 35 Ag_2S devices.

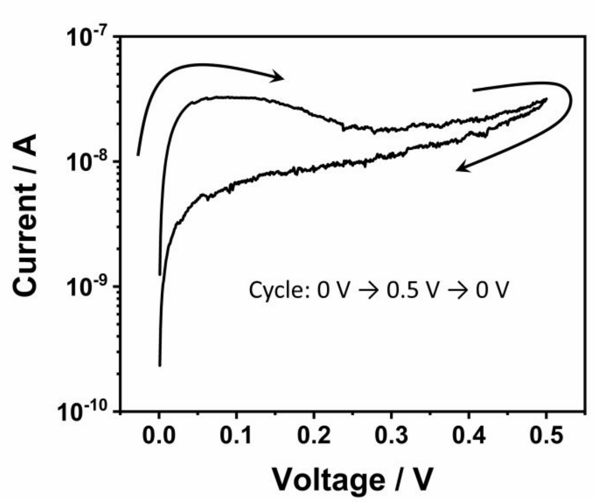

Figure S4. The current-voltage curves recorded by applying electric bias to top electrode of an initial vertical configuration memristor device. Since the device hasn't experienced any setting process, there is no filament inside the Ag_2S electrolyte. However, the recorded I-V curve shows that resistance increases with the applied positive bias, which cannot be explained by the conventional simplified filament ablation process.

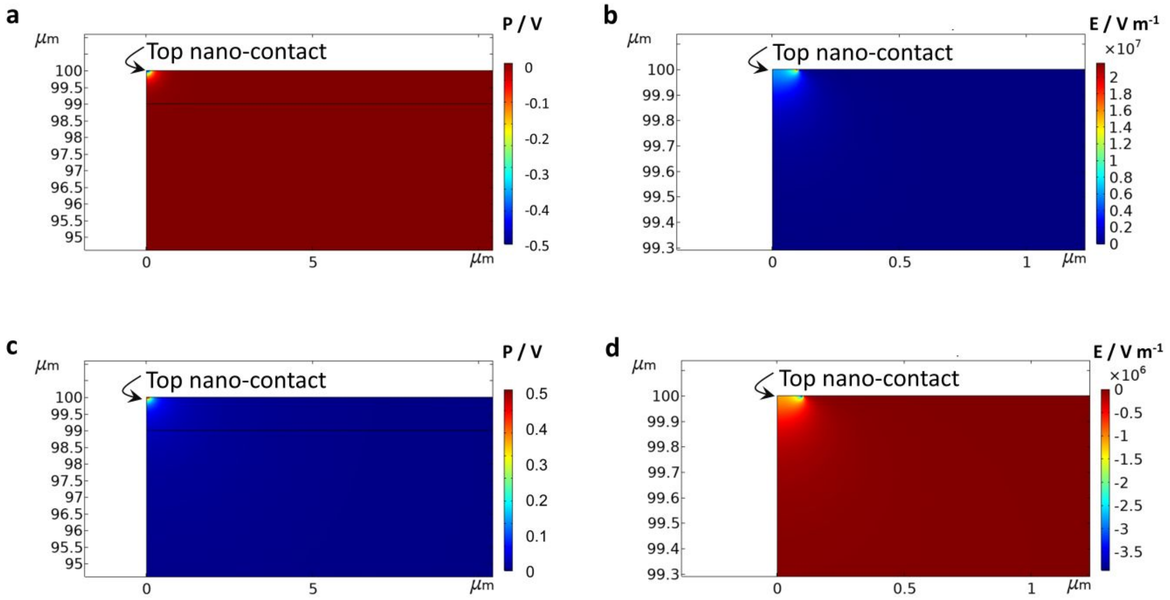

Figure S5. Simulated electric potential and electric field distribution of vertical configuration device using finite element analysis. (a) The potential distribution under -0.5 V bias. The result shows that the top nano-contact interface takes the most of the setting bias. (b) The simulated electric field distribution under a -0.5 V setting bias. The resulted large electric field of about 10^7 V/m at top interface enables effective migration of Ag^+ ions to form silver accumulation and filaments. (c) The potential distribution under 0.5 V bias. When a 0.5 V bias is applied on top, this top Schottky contact is forwardly biased. The top interface remains taking the main applied bias due to its extremely small nano-contact area comparing to the bottom contact, which is responsible for the formation of Ag^+ depletion region. (d) The simulated electric field of device under a 0.5 V resetting bias. The bias was applied on the top electrode.

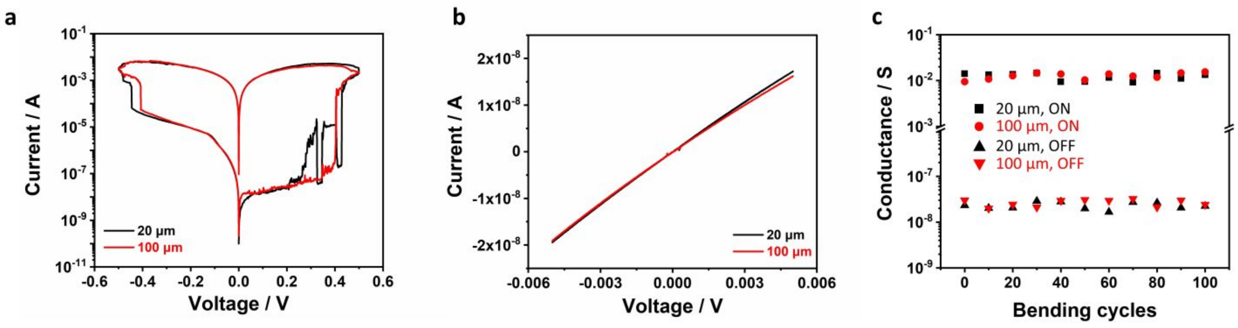

Figure S6. Resistance switching behavior and initial device resistance of memristors fabricated on Ag_2S films with different thickness. (a) Current-voltage curves recorded by scanning voltage from 0 V→ -0.5 V → 0.5 V → 0 V show similar resistance switching behavior of two memristors. (b) The similar initial device resistances of memristors with different Ag_2S thickness indicate the bulk Ag_2S resistance is not dominant in vertical configuration. (c) Conductance variation of devices fabricated (with 100 nm contact hole) using 20 µm or 100 µm Ag_2S films under bending test.

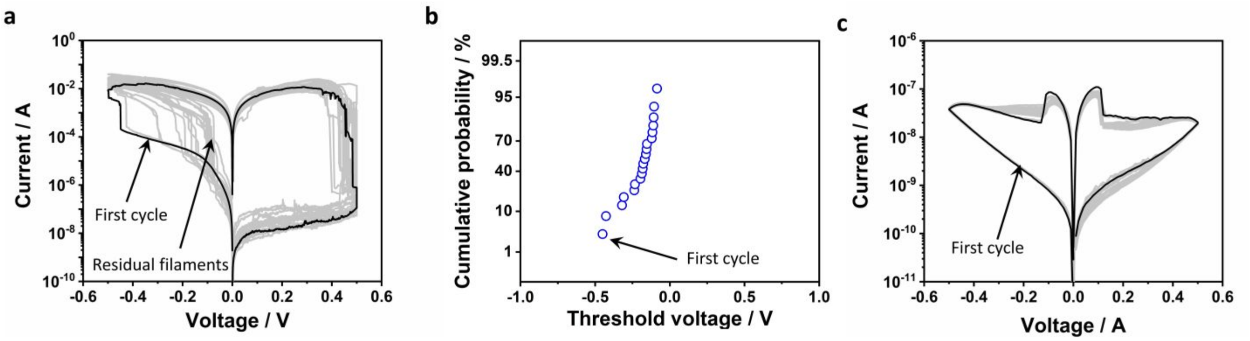

Figure S7. Switching behavior of Ag_2S FM measured in vertical and lateral structure. (a) Repeated current-voltage curves of memristor measured in vertical configuration with 20 repetitive cycles (scanning from 0 V→ -0.5 V → 0.5 V → 0 V). The memristor shows reduced threshold voltage during the following setting process in vertical configuration, which is the characteristic of the

presence of residual filaments after reset. (b) The variations of threshold voltage used to set the device into filament region based on 20 repetitive cycles in Figure S7a. (c) Repeated current-voltage curves of memristor measured in lateral configuration.

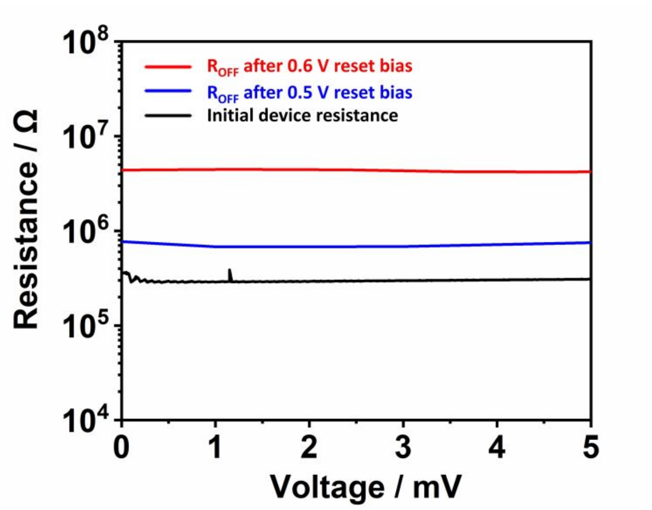

Figure S8. The recorded device resistance at initial and OFF states after 20 ms reset bias pulse. The result shows OFF resistance is higher than initial device resistance and can be further increased by increasing resetting voltage amplitude.

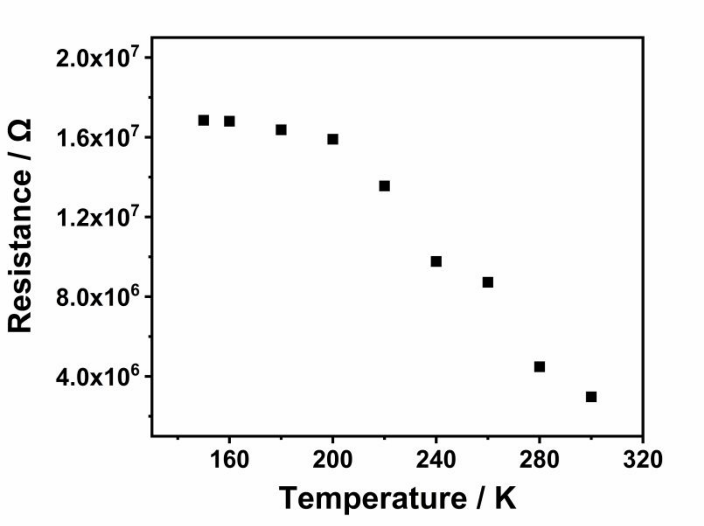

Figure S9. The measured resistance (read at 5 mV) of lateral device after setting at -0.1 V (before current decreasing) under low temperatures. The non-linear resistance dependence on temperature indicates no continuous filament is formed at this point.

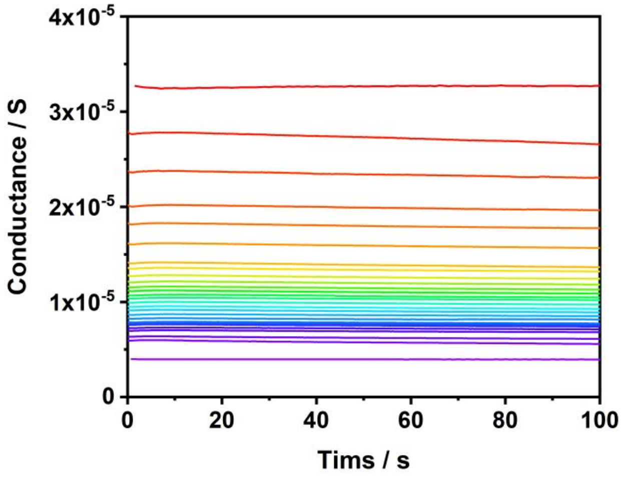

Figure S10 The data retention of 28 conductive states within 3.5×10^{-6} S to 3.5×10^{-5} S in interface resistance switching region.

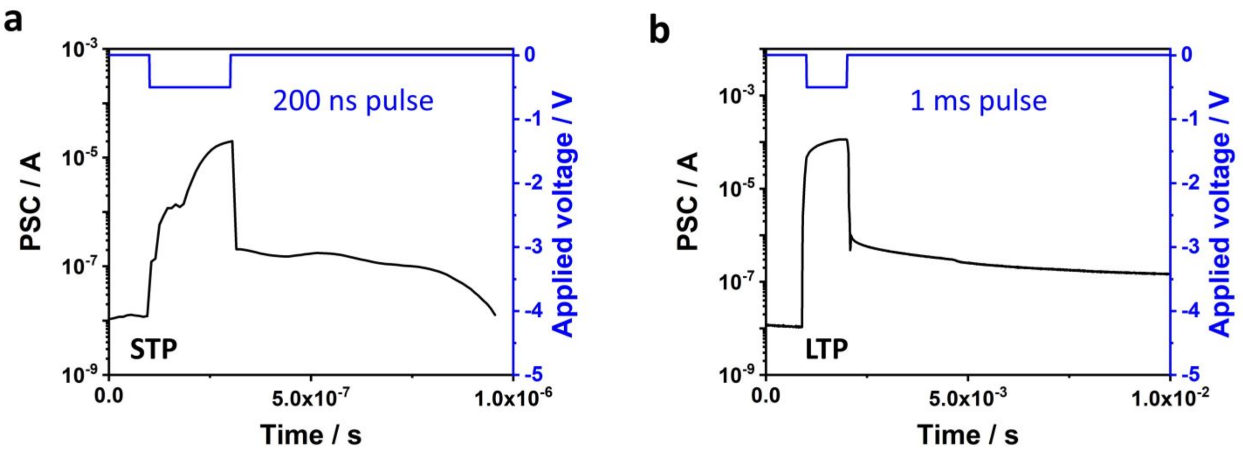

Figure S11 (a) The recorded post-synaptic current (PSC, read at 5 mV) after applying a -0.5 V, 200 ns pulse. The current decays to the initial value in 1 μs, indicating a short-term plasticity. (b) The recorded post-synaptic current (read at 5 mV) after applying a -0.5 V, 1 ms pulse. The higher current maintains its value, indicating a long-term plasticity.
